# Diversity and complexity of the cavotricuspid isthmus in rabbits: A novel scheme for classification and geometrical transformation of anatomical structures

**DOI:** 10.1371/journal.pone.0264625

**Published:** 2022-03-01

**Authors:** Robert Arnold, Ernst Hofer, Josef Haas, Damian Sanchez-Quintana, Gernot Plank

**Affiliations:** 1 Division of Biophysics, Gottfried-Schatz-Research-Center, Medical University of Graz, Graz, Austria; 2 Institute for Medical Informatics, Statistics and Documentation, Medical University of Graz, Graz, Austria; 3 Department of Anatomy and Cell Biology, Faculty of Medicine, University of Extremadura, Badajoz, Spain; Universidad de Zaragoza, SPAIN

## Abstract

The aim of this study was to describe the morphology of the cavotricuspid isthmus (CTI) in detail and introduce a comprehensive scheme to describe the topology of this region based on functional considerations. This may lead to a better understanding of isthmus-dependent flutter and fibrillation and to improved intervention strategies. We used images of the cavotricuspid isthmus from 52 rabbits of both sexes with a median weight of 3.40 ± 0.93 kg. The area of the CTI was 124.25 ± 42.14 mm^2^ with 53.28 ± 21.13 mm^2^ covered by pectinate muscles connecting the terminal crest and the vestibule. Isthmus length decreased from inferolateral (13.09 ±2.14 mm) to central (9.85 ± 2.14 mm) to paraseptal (4.88 ± 1.96 mm) resembling the overall human geometry. Ramification sites of pectinate muscles were identified and six levels dividing the CTI from posterior to anterior were introduced. This allowed the classification of pectinate muscle segments based on the connected ramification level. To account for the high inter-individual variations in size and shape, the CTI was projected onto a normalized reference frame using bilinear transformation. Furthermore, two measures of complexity were introduced: (i) the ramification index, which reflects the total number of muscle segments connected to a ramification site and (ii) the complexity index, which reflects the type of ramification (branching or merging site). Topological analysis showed that the complexity of the pectinate muscle network decreases from inferolateral to paraseptal and that the number of electrically uncoupled parallel pathways increases in the central section between the terminal crest and the vestibule which introduces potential reentry pathways.

## Introduction

The cavotricuspid isthmus (CTI) in the right atrium plays a key role in the formation and maintenance of structure-related rhythm disturbances such as atrial fibrillation (AF) and atrial flutter (AFL). This region is therefore a target for interventional procedures [1–3]. Sequence, success rate, and potential complications of such procedures strongly depend on the anatomical structure of the CTI [4–7]. Hence, detailed knowledge of this region is advantageous for the clinical electrophysiologist.

Within the CTI a rather complex network of cable-shaped pectinate muscles (PM) connects the terminal crest (TC) with the vestibule (VB). From lateral to septal the CTI is bounded by the inferolateral, the central and the paraseptal isthmus (ILI, CI, and PSI) forming a quadrilateral region [6, 8, 9]. From anterior to posterior the CTI is divided by two straight lines into a posterior, middle and anterior sector. This description is well established but characterized by very high inter-individual variability and also the location of the PSI is not consistent throughout literature [6, 8, 10, 11].

In previous studies of human hearts, a classification of the PM network into 6 types according to orientation in relation to the TC, spacing, and branching was introduced [12–14] but to our knowledge no approach takes functional considerations for classification into account: Since the electrical conduction within the CTI is traversing through different anatomical regions, i.e. the TC, the complex network of PMs, and the VB, the activation pattern can be expected to be non-uniform and only piecewise continuous. Hence, waveforms of extracellular potentials recorded clinically with intracardiac catheter electrodes *in-vivo* or during electrophysiological experiments *in-vitro* will strongly depend on the morphology and topology of these conducting substrates in the vicinity of the recording site. Electrophysiological experiments investigating excitation spread and signal waveforms on a structural, microstructural and functional level are often conducted with animal hearts *in-vivo* and *ex-vivo* on isolated whole hearts or partial heart preparations placed in experimental tissue baths [15–17], whereas human studies in this regard are rare for obvious reasons [18, 19]. Animal *in-vitro* experiments are beneficial in two aspects: (i) advanced potential recording methods can be used which are not applicable *in-vivo* (optical mapping with fluorescent dyes, microelectrodes) and (ii) recording sites can be allocated to the underlying anatomical macrostructure with an accuracy unattainable *in-vivo*. In this work we therefore used rabbit atria from studies conducted by our group *post experimentum* for morphometric studies of the CTI to establish a classification scheme based on functional considerations.

The aim of this work is to describe the gross anatomy of the CTI of a large number of rabbit atria (52) as detailed as possible and to develop a system to describe the pathways of conduction, their complexity, and the inter-individual similarities and diversities of the CTI network. For this purpose, a novel morphological and topological classification of the CTI in terms of PM segments and ramification sites was introduced. We propose a more detailed segmentation of PMs from TC towards VB along branching and merging sites with up to six levels of ramification instead of using the current vertical segmentation of the CTI into posterior, middle and anterior sectors along three equidistant straight lines without electro-anatomical correlate.

## Methods

### Tissue preparations

For this work image data acquired from previous electrophysiological studies and projects [20–25] of our group were used (P12293 and P19993 of the Austrian Research Fund, Vienna, Austria). In these projects–which had a different aim– 57 rabbits were weighed, euthanized with an overdose Propofol and Fentanyl and hearts were quickly excised and placed in modified and oxygenated Tyrode’s solution at 5–10°C for tissue preparation. Right atria were dissected, stretched gently and pinned down with needles on a transparent silicone carrier with the endocardium facing up. The carrier with the preparation was placed in a tissue bath and superfused with oxygenated (95% O_2_, 5% CO_2_) Tyrode’s solution at 36.4°C. 5 preparations were excluded from the electrophysiological studies because of damages during preparation (n = 3) and questionable or atypical deformations (n = 2). Images of the remaining 52 preparations were used in this work.

The experimental procedure was performed in accordance to the national ethical standards and regulations (§2 Abs.1 Definition of Animal Procedure, Austrian law TVG 2012 BGBL. I Nr. 114/2012 and §20 Legal Sacrification Methods TVV 2012). All experiments were conducted according to the Directive 2010/63/EU of the European Parliament and of the Council. The rabbit population comprised New Zealand rabbits (Oryctolagus cuniculus) and domestic rabbits (Oryctolagus cuniculus f. domestica) provided by Charles River Laboratories, Sulzfeld, Germany and Center for Biomedical Research, Medical University Vienna, Himberg, Austria.

### Image data

Overview images of the whole right atria were taken at the beginning of the electrophysiological experiments for documentation. 2 different cameras were used: Canon EOS 5D Mark II with Canon EF 180mm f/3.5L USM lens (Canon, Japan) and Olympus compact camera C5060WZ (Olympus, Japan). Depending on the camera and the field of view, a resolution between 6.1 μm/px and 27.8 μm/px was achieved.

Images were taken either with incident light and/or in transillumination. Incident light was provided from a cold light source with flexible light guide (KL 150 B, Schott, Germany) and/or from LEDs arranged in a ring around the edge of the tissue bath. Both light sources allowed contrast enhancement by illumination at a shallow angle. To improve the detection of very thin fiber strands a transillumination device was developed: A LED-array module (GW5BWC15L02 normal white, Sharp, Japan) was mounted in a sink inside a metal case and the case was filled with transparent silicone. This plug-shaped device was placed at the bottom of the tissue bath with the tissue preparation on top [20].

### Determination of the ROI, classification of pectinate muscle segments and ramifications

**Fig 1:**
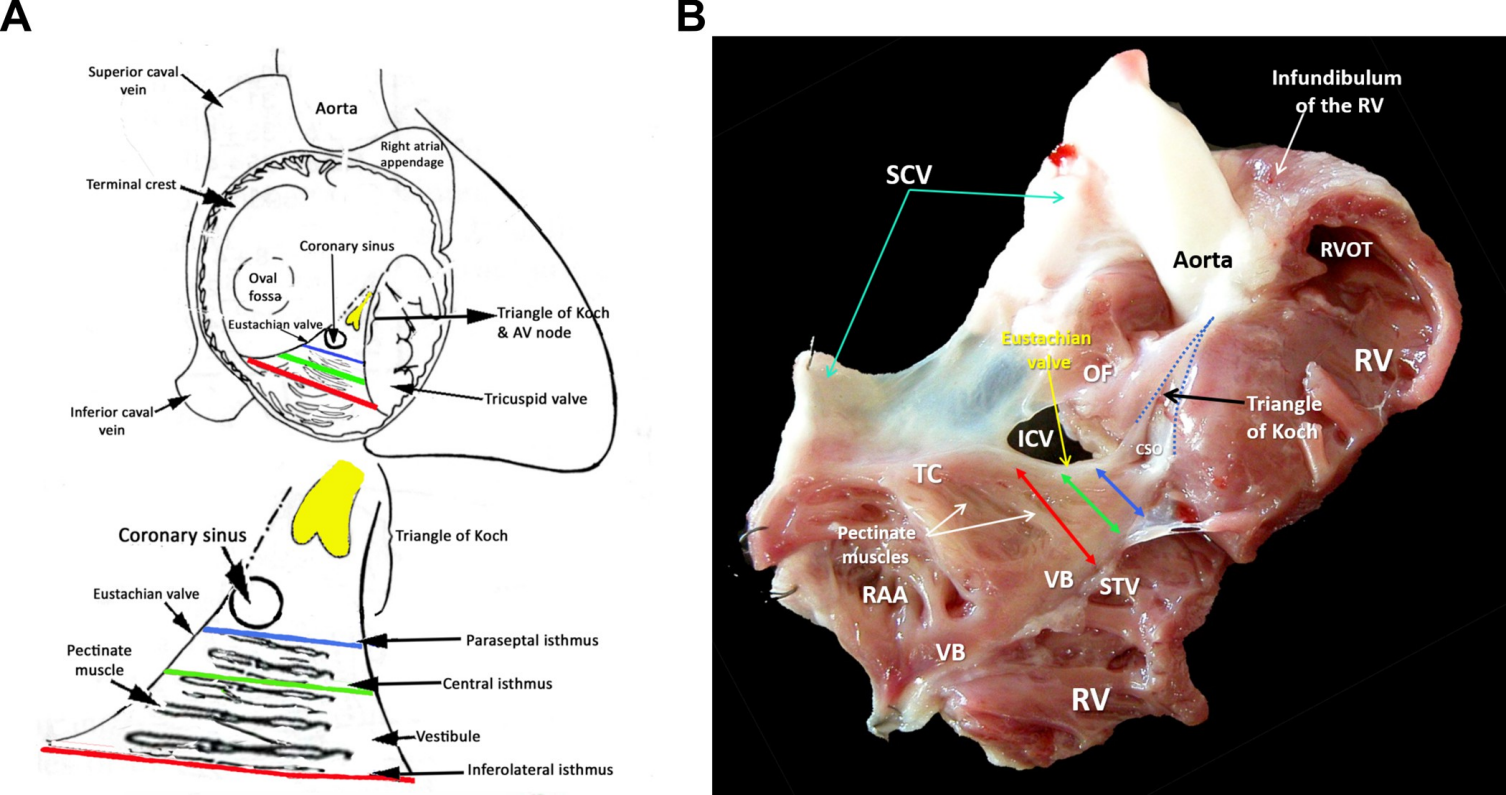
Location of the cavotricuspid isthmus (CTI) within the heart. (A) Illustration of the CTI within the right atrium (Modified with permission from [26]). (B) CTI in a tissue preparation from a rabbit heart. RAA … right atrial appendage, RV … right ventricle, TC … terminal crest, VB … vestibule, SCV … superior caval vein, IVC … inferior caval vein, OF … oval fossa, CSO … coronary sinus ostium, RVOT … right ventricular outflow tract, STV … septal attachment of the tricuspid valve.

The region of interest (ROI), i.e. the CTI, was determined as described in [9–11] by identifying the borders formed by the terminal crest (TC), the vestibule (VB), the paraseptal isthmus (PSI) and the inferolateral isthmus (ILI). Within the CTI lies the central isthmus (CI), dividing the CTI into two lateral parts: the lateral-central and the central-paraseptal part. TC and VB are connected with a network of Pectinate muscles (PMs) which can be seen as a chain of segments between branching and merging sites. The location of the CTI within the heart is illustrated in Fig 1.

**Fig 2:**
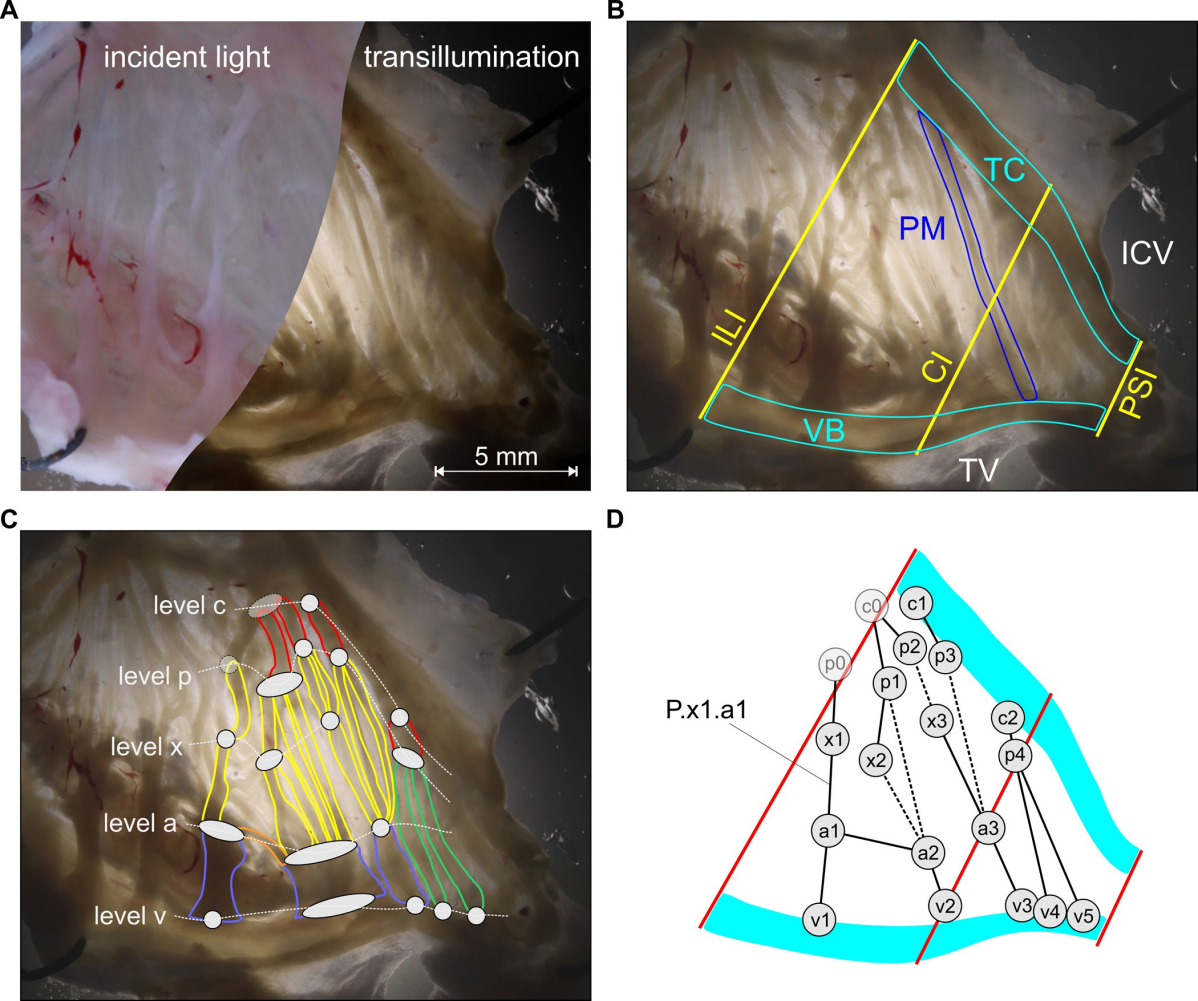
Cavotricuspid isthmus (CTI) and proposed nomenclature for the pectinate muscle network. (A) Image of the specimen with incident light (left) and transillumination (right). (B) The CTI is delimited by the TC, VB, ILI, and PSI. (C) Ramifications of PMs were identified and assigned to levels *c*, *p*, *x*, *y*, *a*, and *v* from TC to VB and numbered from inferolateral to paraseptal. Note that in this particular preparation no level *y* was present. One PM (orange) connected ramifications at the same level, i.e. ramifications R.a1 and R.a2 (D) Abstract representation of the CTI. Solid lines are single PM segments, dashed lines represent multiple parallel connections between ramifications. PM segments are designated by their start- and endpoints, e.g. P.x1.a1. Note that ramifications c0 and p0 are outside of the ROI but are added for labeling of the corresponding PM segments within the CTI. PM … pectinate muscle, TC … terminal crest, VB … vestibule, ILI … inferolateral isthmus, CI … central isthmus, PSI … paraseptal isthmus, TV … tricuspid valve, ICV … inferior caval vein.

For morphometric analysis custom-written software (IEAMS, KMLvision, Graz, Austria) was used. This software provided a database for experiment data and allowed interactive annotations of image data tailored to our needs. Tissue samples were imaged with both incident light (left side of Fig 2A) and transillumination (right side of Fig 2A) as described above. Toggling both image modes in the software facilitated the identification of ramifications and the determination of contours of even very thin PM segments with high resolution. For analysis only PMs clearly located at the endocardial surface with a diameter larger than approximately 100 μm were included.

The branching and merging sites of PMs between the TC and VB were denoted as ramifications (R).

Hence, up to six levels of ramifications were defined depending on the location within the CTI: level *c* comprising ramifications *R*.*c* located at the exit site from the TC (c for Crista Terminalis), level *v* with ramifications *R*.*v* at the entry sites of PMs into the VB (v for Vestibule), level *p* with ramifications *R*.*p* at the first posterior branching site after leaving the TC (p for posterior), and level *a* with ramifications *R*.*a* at the last anterior merging site before reaching the VB (a for anterior). Between *R*.*p* and *R*.*a* further ramifications can be found eventually which we included by introducing sublevels of ramifications *R*.*x* and *R*.*y*. Within one level, ramifications are indexed with integer numbers incrementing from inferolateral to paraseptal, e.g. *R*.*a3* is the third merging site at level *a*. The complete scheme is illustrated in Fig 2.

PM segments connect the ramification sites and form a network of conduction pathways within the CTI. Contours of the PM segments were manually drawn in IEAMS. For each segment area, length, and diameter was evaluated. Length *L* and diameter *D* of the irregular shaped contours were estimated upon the contour area *A* and circumference *C* by solving following equations:

$$L=\frac{C+\sqrt{C^{2}-16\cdot A}}{4}$$ (1)

$$D=\frac{C-\sqrt{C^{2}-16\cdot A}}{4}$$ (2)

This approximation by a rectangular shape is sufficiently accurate for short and/or thin PM segments as they are found in the preparations (compare Fig 1C).

**Fig 3:**
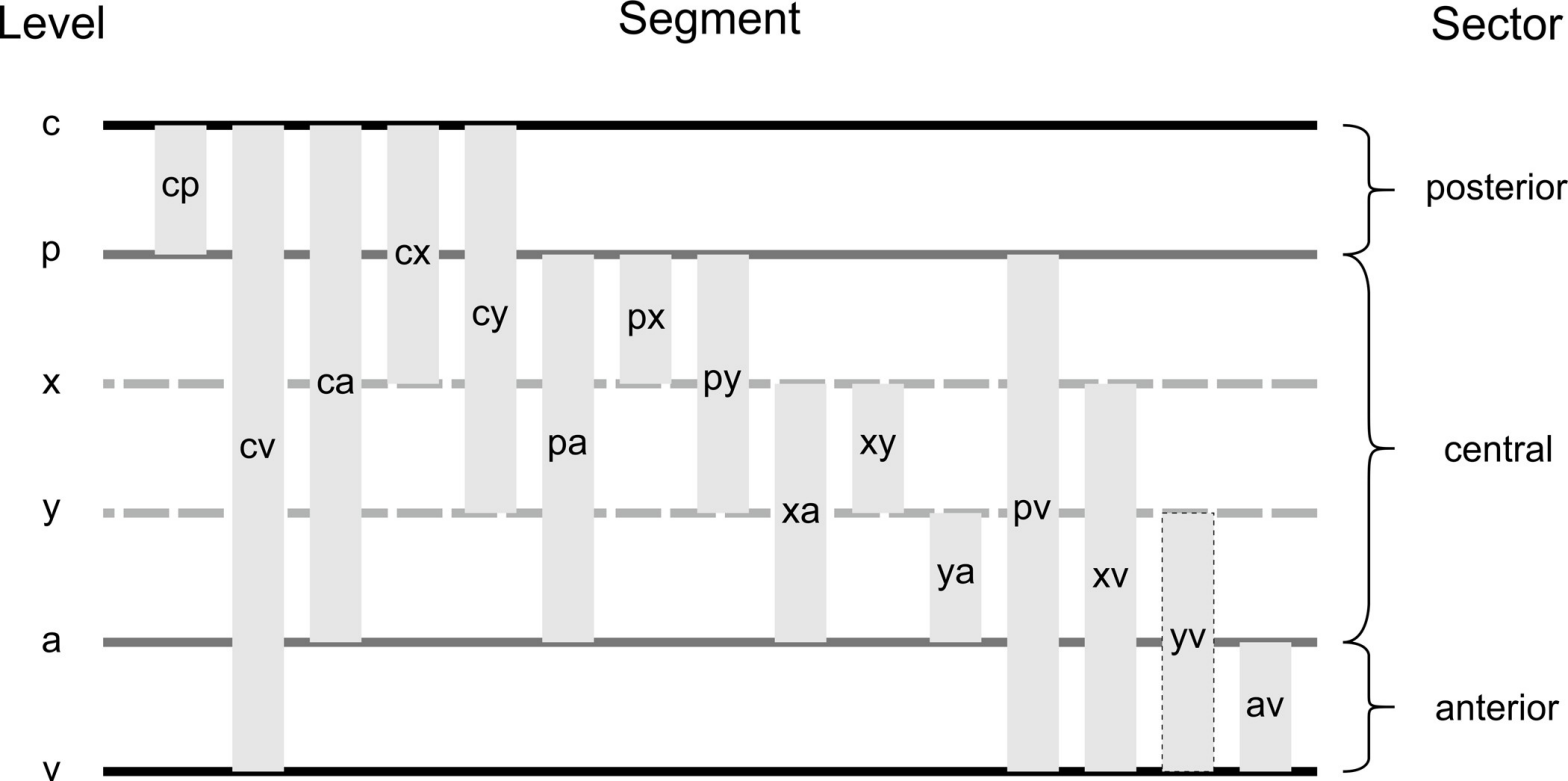
Ramification levels and types of PM segments. Depending on the bridged levels, 15 different types of segments can be assigned. PM segment *yv* was not found in any preparation. Three sectors can be defined: posterior between level *c* and *p*, central between level *p* and *a*, and anterior between level *a* and *v*. Connections between same levels are referred to as type *ii* (not shown here, compare Fig 2C and 2D).

The segments are designated by their start- and endpoints, e.g. a PM segment running between branching site *R*.*p4* and merging site *R*.*a5* is labeled *P*.*p4*.*a5*. Segments can span different levels of ramifications and can therefore be clustered into 15 groups of segments as shown in Fig 3. Segments can also connect ramifications at the same level, e.g. *a-a*, and were pooled together in group *ii* (not shown in Fig 3, see Fig 2C and 2D).

Using this nomenclature, the CTI can be divided into three sectors: (i) posterior between *R*.*c* and *R*.*p*, (ii) central between *R*.*p* and *R*.*a*, (iii) and anterior between *R*.*a* and *R*.*v*.

Some few PM segments differ markedly in direction from the majority of the pectinate ensemble, i.e. they are aligned orthogonal to the PM ensemble. These PM segments are also located rather on the epicardium than on the endocardium and were not included in the analyses.

For each ramification site two measures of complexity are introduced: (i) the ramification index RI which corresponds to the total number of PM segments connected to the ramification site and (ii) the complexity index CXI which describes the difference in numbers of afferent (“incoming”) and efferent (“outgoing”) PM segments at a given ramification.


$$RI=N_{afferent}+N_{efferent}$$ (3)

$$CXI=N_{efferent}-N_{afferent}$$ (4)

Thus, RI is a measure for individual conduction pathways leading to and away from ramifications and CXI describes the type of a ramification, i.e. if the ramification is a point where PM segments merge (CXI is negative), branch (CXI is positive), or where the number of PM segments stays the same (CXI is zero).

### Bilinear transformation

**Fig 4:**
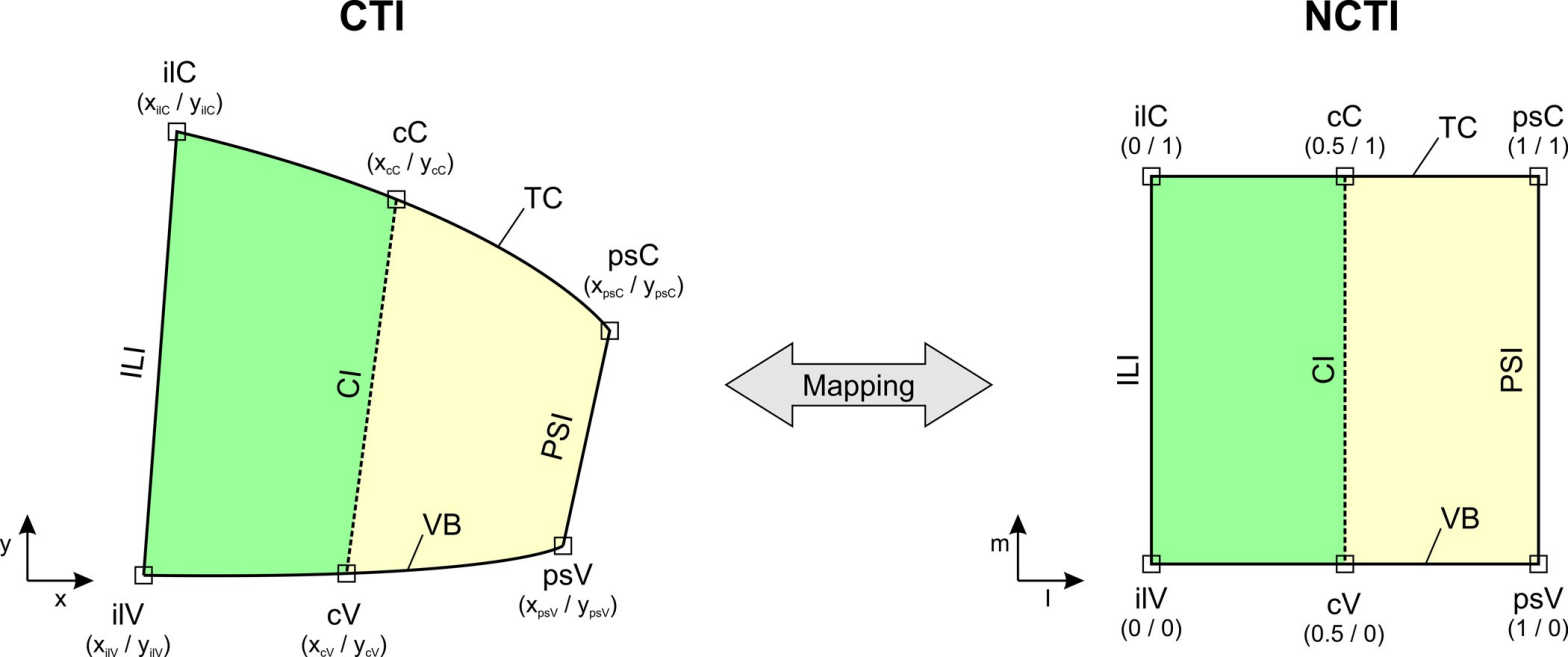
Bilinear transformation from image coordinates to normalized coordinates. The contour of the CTI can be described by 6 points, i.e. the intersections of the 3 isthmi with the TC and the VB (left). These 6 points in image coordinates *x/y* are transformed by a bilinear mapping function onto 6 points of a unit square in normalized coordinates *l/m* where TC and VB are the top and bottom edges, ILI and PSI are the left and right edges and CI divides the unit square into two halves. We refer to the transformed contour as “normalized CTI” (NCTI).

To compare CTI topologies of different specimens, the CTI had to be normalized to account for varying shape and size of the CTI. The CTI is determined by the circumference of TC, PSI, VB, and ILI and is divided in lateral-septal direction into two parts by the CI as described above. These two parts are irregular quadrilaterals sharing the CI as a common border. Therefore, the edges of the left part between ILI and CI and the edges of the right part between CI and PSI were mapped to two adjoining halves of a unit quad with an edge length of 1 as shown in Fig 4. We refer to this transformed ROI as “normalized CTI” (NCTI).

Points within the ROI have to be transformed between the image coordinate system *x/y* and the normalized coordinate system *l/m* and vice versa. Since the coordinates in *l/m*-space are predefined it is easiest to find the required transformation parameters by starting with equations for the transformation from normalized coordinates to image coordinates. The bilinear mapping functions are therefore given by

$$x=\alpha_{1}+\alpha_{2}\cdot l+\alpha_{3}\cdot m+\alpha_{4}\cdot l\cdot m$$ (5)

and

$$y=\beta_{1}+\beta_{2}\cdot l+\beta_{3}\cdot m+\beta_{4}\cdot l\cdot m$$ (6)

To solve for the unknown coefficients, the four corners of each part of the NCTI are used: for the left part the intersection of ILI and CI with TC and VB yields the points *ilV*, *cV*, *cC*, and *ilC* and for the right part the intersection of CI and PSI with TC and VB yields the points *cV*, *psV*, *psC*, and *cC*. Inserting the given coordinates in *l/m*-space yields for example following equation for the x-coordinates of the corners of the left CTI quadrilateral:

$$\left[\begin{array}{c} x_{ilV}\\ x_{cV}\\ x_{cC}\\ x_{ilC} \end{array}]=[\begin{array}{cccc} 1 & 0 & 0 & 0\\ 1 & 0.5 & 0 & 0\\ 1 & 0.5 & 1 & 0.5\\ 1 & 0 & 1 & 0 \end{array}][\begin{array}{c} \alpha_{1,left}\\ \alpha_{2,left}\\ \alpha_{3,left}\\ \alpha_{4,left} \end{array}\right]$$ (7)

Similar expressions are obtained for the y-coordinates of the left quadrilateral and the x/y-coordinates of the right quadrilateral resulting in 2 sets of coefficients α and β. Solving for the coefficients α and β is done by inverting the matrices and inserting the determined points in image coordinates, e.g.


$$\left[\begin{array}{c} \alpha_{1,left}\\ \alpha_{2,left}\\ \alpha_{3,left}\\ \alpha_{4,left} \end{array}]=[\begin{array}{cccc} 1 & 0 & 0 & 0\\ -2 & 2 & 0 & 0\\ -1 & 0 & 0 & 1\\ 2 & -2 & 2 & -2 \end{array}][\begin{array}{c} x_{ilV}\\ x_{cV}\\ x_{cC}\\ x_{ilC} \end{array}\right]$$ (8)

For mapping from image coordinates to normalized coordinates, Eqs (5) and (6) have to be solved for *l* and *m*:

$$l=\frac{x-\alpha_{1}-\alpha_{3}\cdot m}{\alpha_{2}+\alpha_{4}\cdot m}$$ (9)

$$\begin{array}{c} 0=(\alpha_{4}\beta_{3}-\alpha_{3}\beta_{4})\cdot m^{2}\\ +(\alpha_{4}\beta_{1}-\alpha_{1}\beta_{4}+\alpha_{2}\beta_{3}+\alpha_{3}\beta_{2}+x\cdot \beta_{4}-y\cdot \alpha_{4})\cdot m\\ +(\alpha_{2}\beta_{1}-\alpha_{1}\beta_{2}+x\cdot \beta_{2}-y\cdot \alpha_{2}) \end{array}$$ (10)

Eq (10) is a simple quadratic equation with the solution

$$m=\frac{-b+\sqrt{b^{2}-4ac}}{2a}$$ (11)

Hence, mapping of any point in image coordinates *x/y* to coordinates *l/m* in “unit space” and vice versa can be performed.

### Histology

To depict the microstructure in the subregions of the CTI, one atrial preparation (not included in the quantitative evaluation in this work) was fixed in 7% neutral buffered formaldehyde, dehydrated and embedded in paraffin. The paraffin block was serially sectioned parallel to the septal leaflet of the tricuspid valve using a microtome (Microm HM310, Thermo Fisher Scientific, Walldorf, Germany) with a sectioning width of 7 μm. Every 10th section was used for staining with Masson’s trichrome which labels connective tissue bluish-green, myocytes pinkish-red and nuclei blue-black. Cleft spaces remain unstained and appear white in the images. Micrographs were then digitized (Scan Scope, Aperio, Vista CA, USA) in SVS file format (20 000 × 35 762 pixels).

### Statistics

Data were tested for normal distribution using Shapiro-Wilk test. For normally distributed data values are given as mean ± standard deviation (SD). If normal distribution was rejected, values are given as median ± median absolute deviation (MAD). Groups were compared with Mann-Whitney rank tests, because data was either not normally distributed or sample size was too small for parametric testing. A p-value of 0.05 was considered to be statistically significant. Statistical analyses and plotting were performed in Python programming language (Python Software Foundation, version 3.7.9. Available at http://www.python.org) using the packages scipy [27], seaborn [28], and matplotlib [29].

## Results

### Specimen

Rabbits of both sexes were used in the studies. The population comprised 21 females (40%), 23 males (44%), and 8 rabbits with undocumented sex (15%). Regarding species, 11 New Zealand rabbits (6 female, 5 male) and 41 domestic rabbits (15 female, 18 male) were included. Morphometric parameters of both types of rabbits were compared. Statistically significant differences were only found in weight (New Zealand rabbits were purchased at a younger age in the underlying study) and in diameter and area of the TC (these morphological features were analyzed in Table 2 but play no role in the remaining evaluation of the CTI). Results are shown in S1 Fig. Therefore, both types of rabbits are pooled together for further analysis.

Body weight of rabbits was not normally distributed (p-value 0.024) with a weight of 3.40 kg ± 0.93 kg (median ± MAD) and a range of 1.80 kg—6.20 kg. There was no statistically significant difference in body weight between both sexes (p-value 0.49). Specimens included in the study are summarized in Table 1.

**Table 1:** Summary of included specimens.

		#	body weight (kg)	range (kg)
**NZ**	total	11	2.75 ± 0.31	2.53–6.20
	male*	5	2.55 ± 0.02	2.53–2.58
	female	6	3.00 ± 0.33	2.75–6.20
**Dom.**	total*	41	3.85 ± 0.95	1.80–6.05
	male*	18	3.74 ± 0.83	2.15–6.05
	female*	15	3.82 ± 0.92	2.45–5.90
	unknown*	8	2.76 ± 0.79	1.80–3.85
**Overall**	total	52	3.40 ± 0.93	1.80–6.20
	male*	23	3.48 ± 0.88	2.15–6.05
	female	21	3.47 ± 0.99	2.45–6.20
	unknown*	8	2.76 ± 0.79	1.80–3.85

NZ … New Zealand rabbits, Dom. … Domestic rabbits.

* normal distribution (p = 0.05). Body weight for normally distributed data is given as mean ± SD and as median ± MAD otherwise.

### Morphometry

For each of the 52 atrial preparations areas, lengths and diameters of the CTI and its subregions were determined (Table 2). The area within the CTI which is covered by PM segments (CTI_m) was 42.68% ± 8.31% and the non-muscular area (CTI_nm), i.e. the total CTI area minus the area of all PM segments, TC, and VB, was 29.98% ± 7.61%. The ratio of non-muscular to muscular area (CTI_nm_ratio) was 65.87% ± 25.07%.

**Table 2:** CTI morphology.

	CTI_A*	CTI_m*	CTI_nm	CTI_nm_ratio	ILI_L*	CI_L*	PSI_L*	TC_A*	TC_L*	TC_D*	VB_A	VB_L*	VB_D
	mm^2^	mm^2^	mm^2^	%	mm	mm	mm	mm^2^	mm	mm	mm^2^	mm	mm
**mean**	124.25	53.28	31.95	65.87	13.09	9.85	4.88	16.25	13.64	1.19	15.18	12.04	1.30
**±**	±	±	±	±	±	±	±	±	±	±	±	±	±
**SD**	42.14	21.13	14.65	25.07	2.14	2.14	1.96	5.46	3.11	0.27	6.91	2.83	0.46
**min**	43.98	15.12	8.84	19.06	8.65	5.31	1.67	4.82	6.91	0.62	4.97	5.63	0.65
**max**	217.28	102.40	89.14	184.47	18.77	14.79	8.75	30.18	19.90	1.87	50.14	17.01	3.68

CTI_A … total CTI area, CTI_m … muscular area within CTI, CTI_nm … non-muscular area within CTI, CTI_nm_ratio … ratio of non-muscular to muscular area within CTI, ILI_L … length of inferolateral isthmus, CI_L … length of central isthmus, PSI_L … length of paraseptal isthmus, TC_A, TC_L, TC_D … area, length, and diameter of TC, VB_A, VB_L, VB_D … area, length, and diameter of VB.

* normal distribution (p = 0.05). Values for normally distributed data are given as mean ± SD and as median ± MAD otherwise.

**Fig 5:**
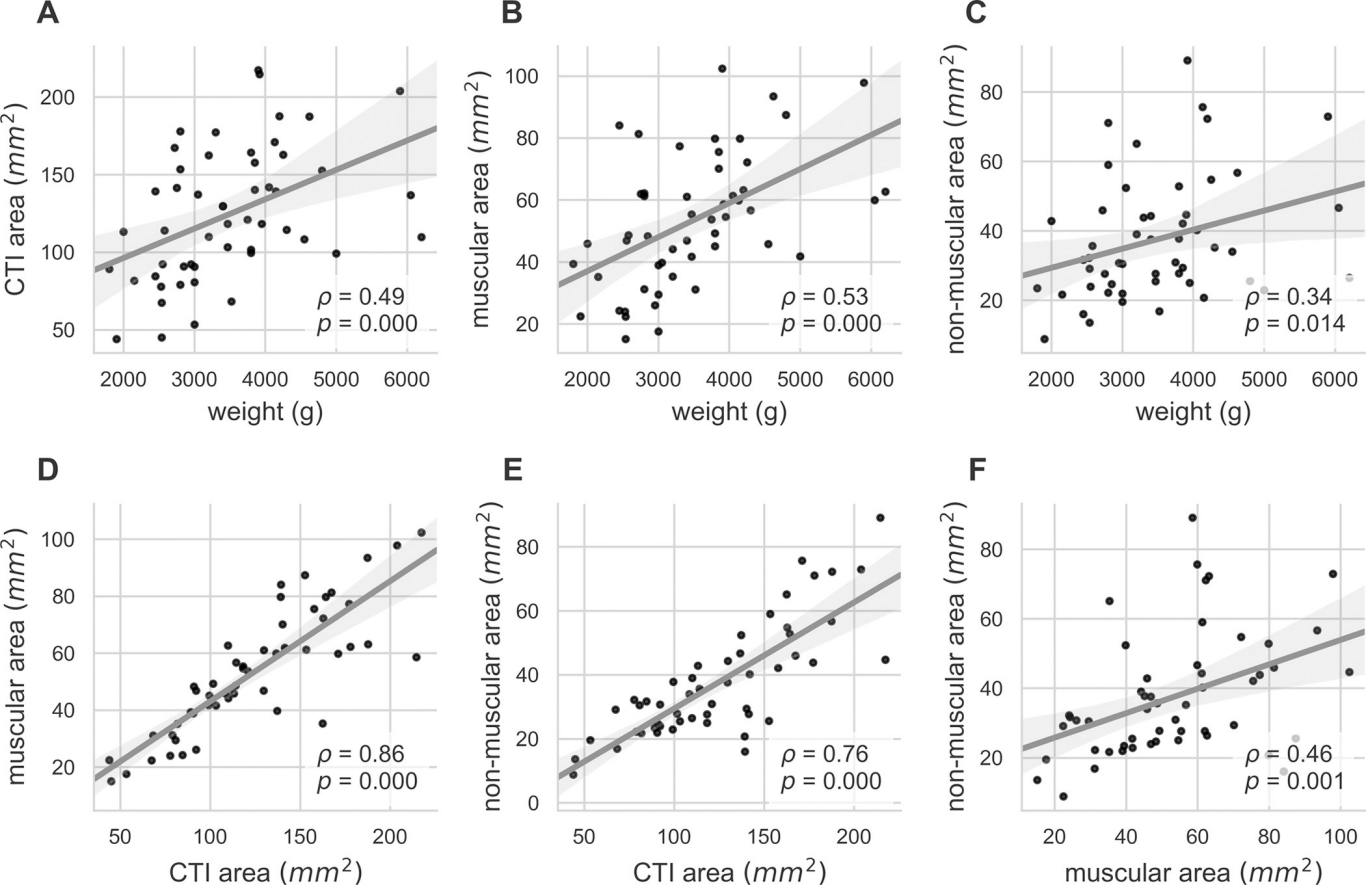
Morphometric analyses of the CTI. (A), (B), (C) Total area of the CTI, muscular area, and non-muscular area are increasing with animal weight. (D), (E) Muscular and non-muscular area within the CTI is increasing with total CTI area. (F) Non-muscular area is increasing with muscular area at a rate of 0.35 within the CTI. For all panels linear regression was performed (grey line) and Spearman’s *ρ* with associated p-value is given. Grey area shows the 95% regression confidence interval.

Exploratory analysis of relationship between morphometric features in Table 2 was done by scatter plots and linear regression shown in Fig 5. The total area of the CTI, the muscular area, and the non-muscular area are increasing with animal weight (Spearman correlation coefficient *ρ* = 0.49 *ρ* = 0.53, and *ρ* = 0.34 respectively). The amount of muscular area and non-muscular is increasing with the total CTI area (*ρ* = 0.86 and *ρ* = 0.76 respectively) and the non-muscular area is increasing with muscular area (*ρ* = 0.46).

Regarding its shape, the CTI is composed of two trapezoidal shaped parts with isthmus lengths decreasing from inferolateral to paraseptal. Ratios of the three isthmus lengths are given in Table 3.

**Table 3:** Trapezoidal shape of the CTI.

	CI / ILI	PSI / CI*	PSI / ILI*
	%	%	%
**mean ± SD**	75.37 ± 11.19	49.47 ± 17.23	37.29 ± 14.02
**min**	53.68	20.74	15.68
**max**	121.81	107.45	75.21

ILI … inferolateral isthmus, CI … central isthmus, PSI … paraseptal isthmus.

* normal distribution (p = 0.05). Values for normally distributed data are given as mean ± SD and as median ± MAD otherwise.

3 CTIs differed from the usually observed decremental isthmus length ILI > CI > PSI: 2 ROIs had a larger CI than ILI and one ROI had a larger PSI than CI. For all CTIs PSI was smaller than ILI.

A total of 908 PM segments were identified. 52 PMs located rather at the epicardium with orthogonal orientation were excluded. For each bridging level as described in Fig 3 area, length and diameter were calculated. Results are shown in Table 4. 24 PM segments were bridging nodes at the same level, e.g. level *p*—level *p*. These segments are consolidated in column *P*.*ii*.

**Table 4:** Morphometric analyses of area, length and diameter of PM segments.

	P.cp	P.cv	P.ca	P.cx	P.cy	P.pa	P.px	P.py	P.xa	P.xy	P.ya	P.pv	P.xv	P.yv	P.av	P.ii
N	119	39	80	4	1	174	92	6	102	18	15	6	2	0	174	24
**area A (mm^2^)**																
**Median**	1.9	4.2	2.6	0.9	1.9	2.0	1.1	1.3	1.4	0.9	1.1	4.3	2.8		2.9	1.0
**MAD**	1.0	2.0	1.2	0.5		1.2	0.8	0.7	0.7	0.7	0.9	0.8			1.6	0.8
**Min**	0.2	1.7	0.7	0.4		0.4	0.3	0.7	0.3	0.4	0.5	3.2	4.3		0.4	0.2
**Max**	5.8	11.5	13.0	4.5		5.3	3.9	2.9	5.1	3.1	3.8	5.0	1.3		12.4	5.6
**length L (mm)**																
**Median**	3.2	6.0	5.9	3.1	3.5	4.4	2.6	3.6	2.9	2.3	1.9	6.2	5.3		3.2	2.4
**MAD**	1.1	2.5	2.0	2.0		1.7	1.1	0.6	1.1	1.0	1.3	0.5			1.1	1.1
**Min**	0.7	2.4	1.5	1.7		1.5	0.8	2.3	0.8	1.4	0.9	5.8	6.7		0.8	1.0
**Max**	7.2	11.2	9.7	4.4		9.3	6.5	5.7	6.9	4.8	3.5	8.1	3.9		7.3	5.1
**diameter D (mm)**																
**Median**	0.6	0.8	0.5	0.3	0.6	0.5	0.4	0.5	0.5	0.4	0.6	0.7	0.5		0.9	0.4
**MAD**	0.2	0.3	0.2	0.1		0.2	0.2	0.1	0.2	0.1	0.3	0.1			0.3	0.2
**Min**	0.2	0.4	0.3	0.2		0.2	0.2	0.2	0.2	0.3	0.3	0.5	0.6		0.4	0.1
**Max**	1.7	1.3	1.4	1.0		1.2	1.2	0.5	1.2	1.1	1.2	0.8	0.3		2.1	1.1

All values are given as Median ± MAD.

**Fig 6:**
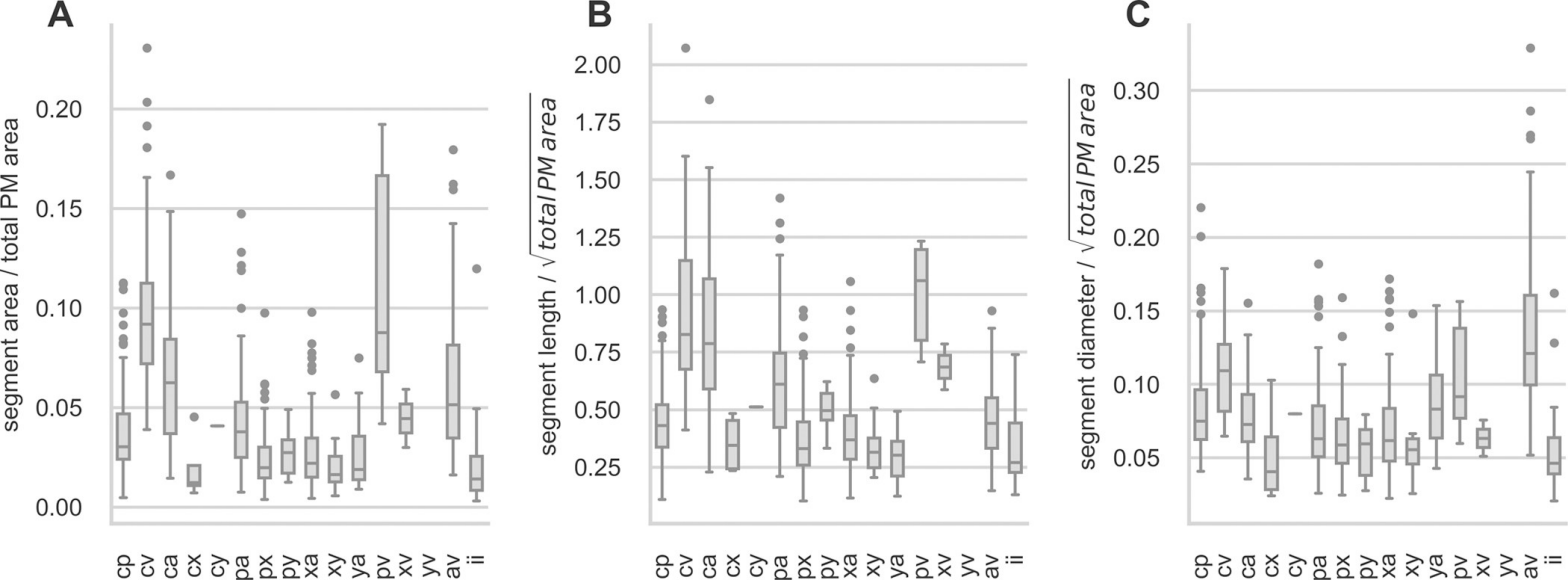
Distribution of morphological parameters grouped by PM segment type. (A) area of PM segments, (B) length of PM segments, (C) diameter of PM segments. Segment area was normalized to total muscular area within the CTI and length and diameter were normalized to the square root of total muscular area.

For boxplotting and pairwise comparison of PM segments, values were scaled as follows: PM area was normalized to the total muscular area within the CTI, PM length and diameter were normalized to the square root of total muscular area to account for the quadratic relation of length and area. Boxplots are shown in Fig 6.

**Fig 7:**
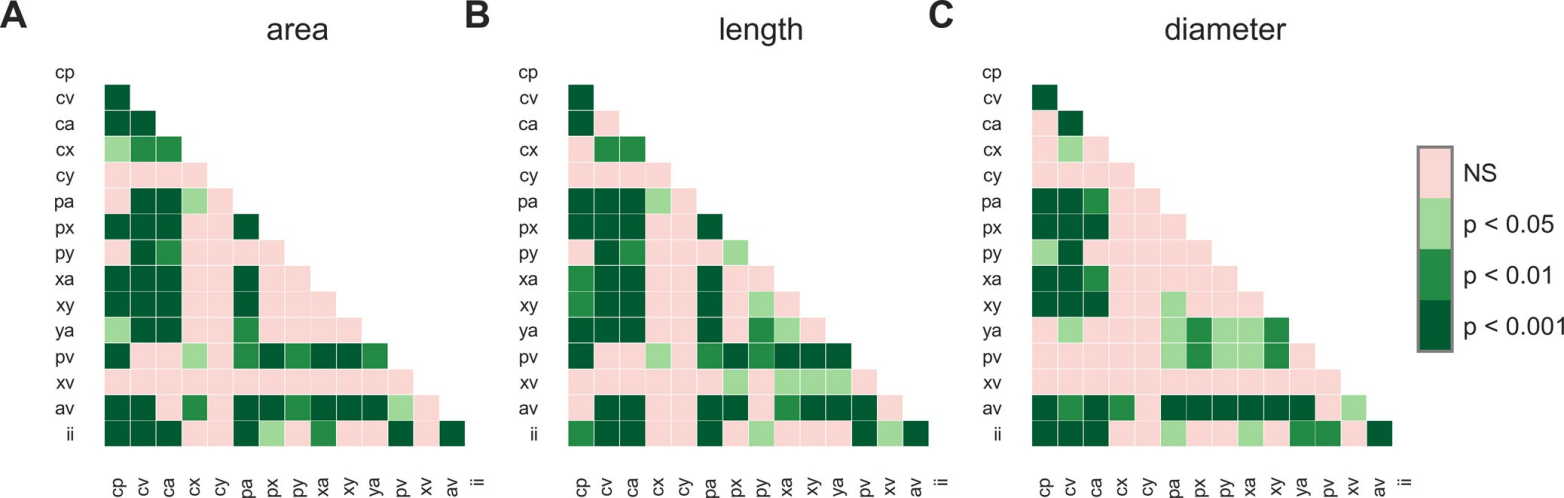
Pairwise comparison of scaled area, length, and diameter for PM segments. Each box shows the p-value of a two-sided Mann-Whitney-U test with a p-value of 0.05 considered as statistically significant. NS … not significant.

For exploratory analysis and illustration scaled area, length and diameter of PM segments were pairwise compared using a two-sided Mann-Whitney-U test without adjustment of p values. Results are shown in Fig 7.

Summarizing Figs 6 and 7, and Table 4 the most apparent observations are: (i) Short but thick PM segments are found for connections between levels *a-v*, i.e. “stems” in the anterior sector. (ii) The largest areas are found for segments *cv* and *pv*. These segments are also the longest and have average diameters. (iii) Short and thin segments are found in the central sector.

### Topology

Within the ROI of all 52 specimens a total number of 832 ramification sites were identified. Topology showed a large degree of variation in complexity with the number of ramification sites within the CTI ranging from 7 to 29. For each specimen the ratio of ramifications at level *c* and level *v* was calculated to compare the number of sites where excitation enters the PM network to the number of sites where excitation ends. Numbers of ramifications are given in Table 5. Note that there is one CTI with no ramifications at level *c*, i.e. *R*.*c* is zero. Here the only ramification from the TC lies outside the CTI and is therefore not counted.

**Table 5:** Ramifications within the CTI.

	total	R.c	R.p	R.x	R.y	R.a	R.v	R.c / R.v
**median**	16	4	3	1	0	3	4	1
**IQR**	6	2	2	2	0	2	2	1
**min**	7	0	0	0	0	1	2	0
**max**	29	8	5	7	2	7	8	2

Complexity of the network within the CTI is highly diverse, with the number of ramifications ranging from 7 to 29. Note the minimum number of ramifications *R*.*c*, i.e. “starting points” in the TC, being zero. This results from one specimen where the only ramification at the TC lies outside the CTI. IQR … interquartile range.

**Fig 8:**
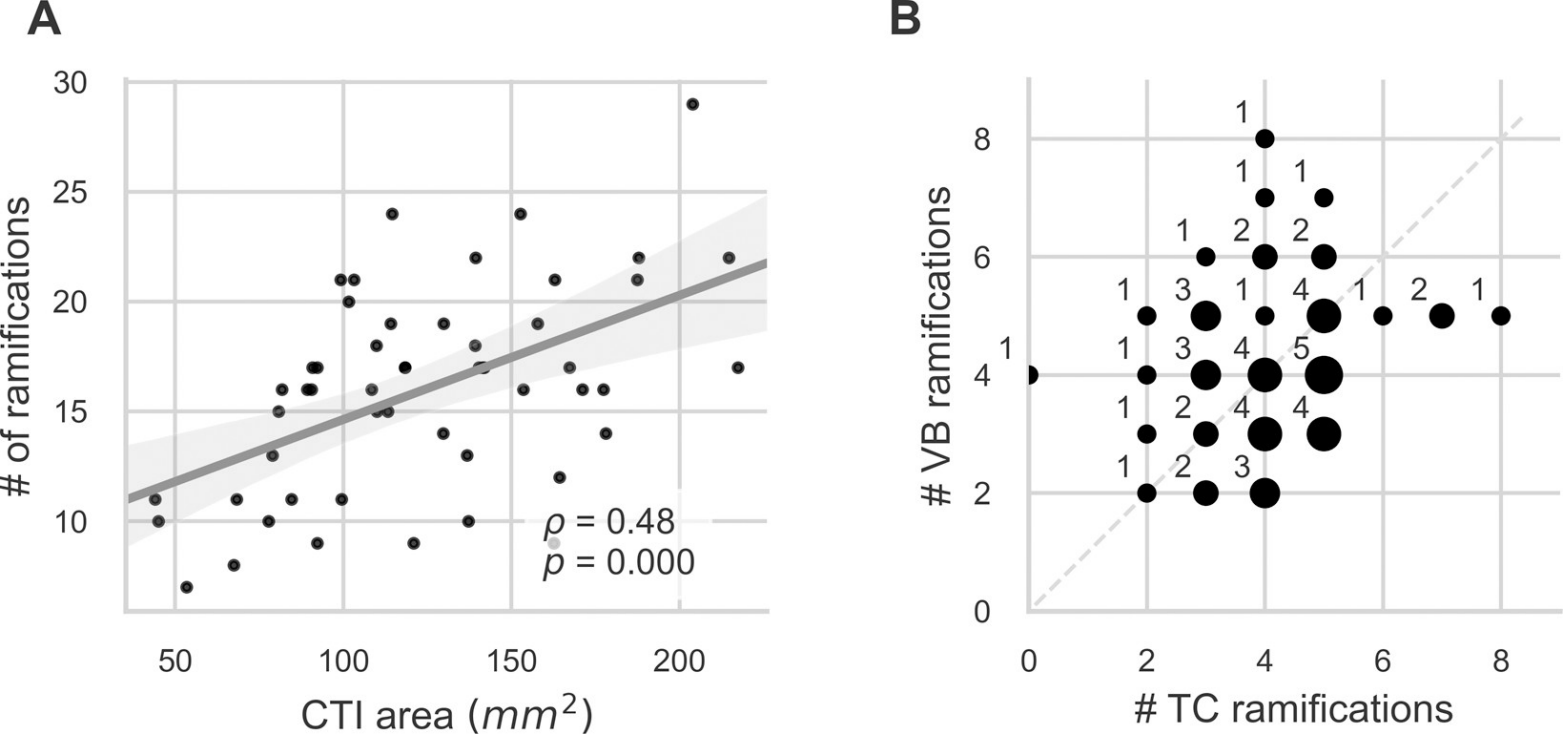
General ramification topology of the CTI. (A) Number of ramifications within the CTI versus total CTI area shows an increasing relationship with larger CTIs containing more ramifications. (B) Ramifications in the TC (starting sites) versus ramifications in the VB (ending sites). Marker area is proportional to number of observations, number of observations is additionally given next to markers. In (A) linear regression was performed (grey line) and Spearman’s *ρ* with associated p-value is given. Grey area shows the 95% regression confidence interval.

The number of ramifications within the CTI is increasing with CTI area as shown in Fig 8A. Concerning the number of starting points of PM segments leaving the TC and ending points where PM segments reach the VB, Fig 8B shows that 11 specimens had the same number of starting points *R*.*c* and ending points *R*.*v*. Slightly more specimens had more *R*.*c* than *R*.*v* (22, below the 1. median) as vice versa (19, above 1. median).

The number of PM segments crossing the three sectors of the CTI (posterior between level *c* and *p*, central between level *p* and *a*, and anterior between level *a* and *v*; compare Fig 3) reflects the overall branching topology. The posterior sector is crossed by 4 (2, 1–9) PM segments, the central sector is crossed by 10 (2, 4–23) PM segments, and the anterior sector is crossed by 4 (2, 2–8) PM segments. Values are given as median (IQR, min—max). This shows that the number of electrically uncoupled pathways is increasing markedly in the central sector, a circumstance relevant for sustaining arrhythmias by introducing potential reentry pathways.

**Fig 9:**
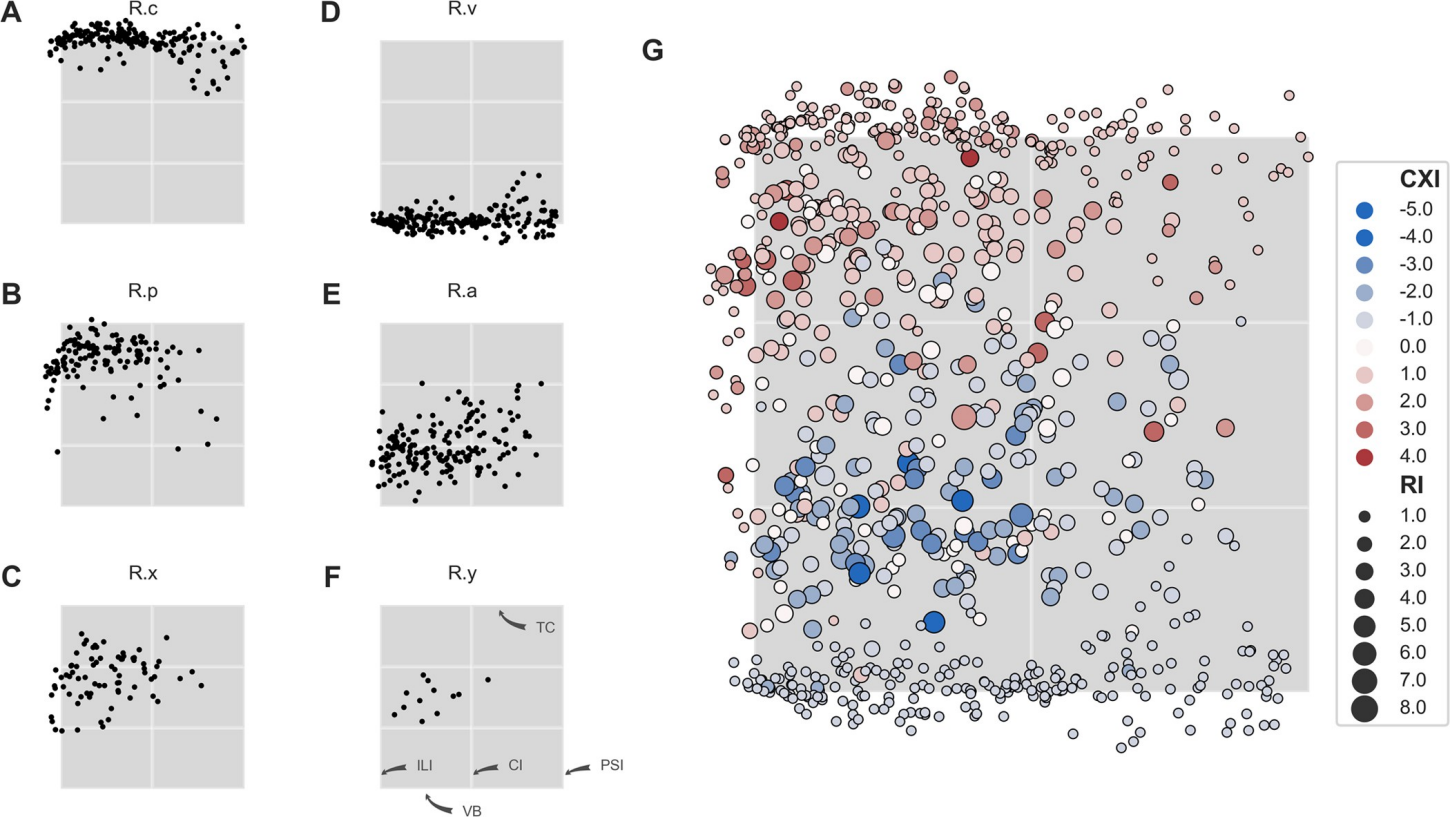
Location and complexity of ramifications within the NCTI. Grey box in all panels shows the NCTI. For better visual orientation, the NCTI is divided by white lines into three vertical domains of equal size and into left and right part. (A)-(F) Location of ramifications within the NCTI grouped by ramification level *c*—*v*. (G) Distribution of ramification index RI and complexity index CXI within the NCTI. CXI is color coded and marker area corresponds to RI. Ramifications with large RI and negative CXI (merging sites) are located between 20% and 50% of the CTI height mainly in the left half. Ramifications with large RI and positive CXI (branching sites) are located between 60% and 90% of the CTI height, again mainly in the left half. In (F) the anatomical correlate of the edges and the center line is shown (compare Fig 4).

To compare topology between specimens, bilinear transformation was performed for each specimen, i.e. the CTI area was normalized to a unit square NCTI. Location of the ramifications within the NCTI is shown in Fig 9A–9F.

Location of the ramifications in Fig 9 is often outside of the unit area, especially for *R*.*c* and *R*.*v* since the curvature of the TC and VB is mapped onto straight lines. Starting points in the TC are clustered at the top edge of the unit square as expected, with a decreasing frequency as the PSI is approached. The same applies for ending points in the VB. First branching of segments at *R*.*p* occurs most frequently in the upper third of the inferolateral half of NCTI. Note the numerous ramifications *R*.*p* and *R*.*x* outside of the unit square, which actually are branching sites outside of the NCTI but are linked by PM segments to ramifications within the NCTI and are therefore included in the analysis. Similar observations are made for the ramifications *R*.*a* and the corresponding PM segments entering the VB which occur most frequently in the inferolateral half of NCTI. Ramifications at levels *R*.*x* and *R*.*y* are also most frequently observed in the inferolateral half clustered around a center line between TC and VB. Complexity of ramifications is shown in Fig 9G. Branching points, i.e. ramifications with large RI and CXI larger than 1, are located predominantly at approximately 75% of the NCTI height. Merging points, i.e. ramifications with large RI and CXI lower than -1, are located predominantly at approximately 30% of the NCTI height. Frequency of both, merging and branching sites, decreases from inferolateral to paraseptal.

**Fig 10:**
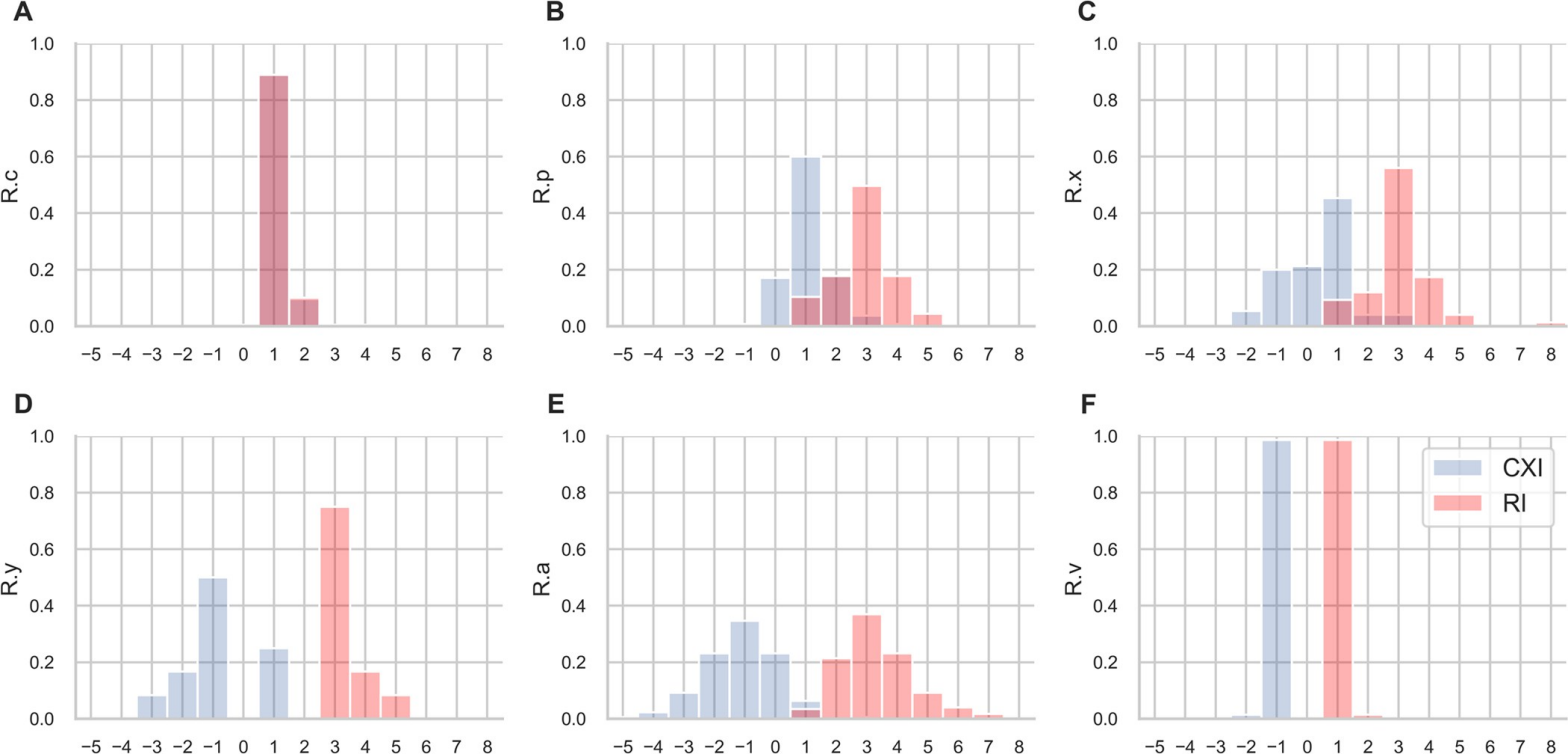
Distribution of CXI and RI. Histogram bars show the probabilities of RI and CXI, i.e. counts are normalized so that the sum of bar heights equals 1. Analysis was performed on ramification levels *c* to *v*. CXI and RI are identical for ramification level *c* (A) as there are no afferent PM segments. CXI and RI at level *v* (F) have identical height but reversed signs as there are no efferent PM segments. CXI range was [-5:4], RI range was [1:8].

For each ramification level probabilities of RI and CXI values are shown as histograms in Fig 10. At level *R*.*c* CXI equals RI as there are no afferent PM segments. The majority of ramifications at this level have a CXI and RI of 1, showing that transitions from the TC into the PM network is carried by discrete, unbranched ramifications. At level *R*.*p* most ramifications show a CXI of 1 and RI of 3, indicating that branching into two parallel conduction pathways occurs. If level *R*.*x* is present, these conduction pathways either further divide into parallel segments (CXI = 1, RI = 3, 45% of ramifications) or complexity decreases when PM segments from different ramifications at level *R*.*p* merge (CXI = -1, RI = 3, 20% of ramifications). At level *R*.*y*, if present, complexity of the PM network starts to decrease with the majority of ramifications showing a CXI of -1 and RI of 3, i.e. two parallel segments merge into one efferent segment. Complexity further decreases at *R*.*a* with the majority of ramifications showing a CXI of -1 and less. At level R.v CXI and RI have equal probabilities but reversed signs as there are no efferent PM segments. Almost all ramifications at this level show a CXI of -1 and RI of 1, indicating that transition from the PM network into the VB is carried by single “stems”.

Summarizing, the complexity of the CTI in terms of density and number of ramification sites and of afferent and efferent PM segments, is highest in the central sector of the inferolateral half of the NCTI and decreases when approaching the PSI.

**Fig 11:**
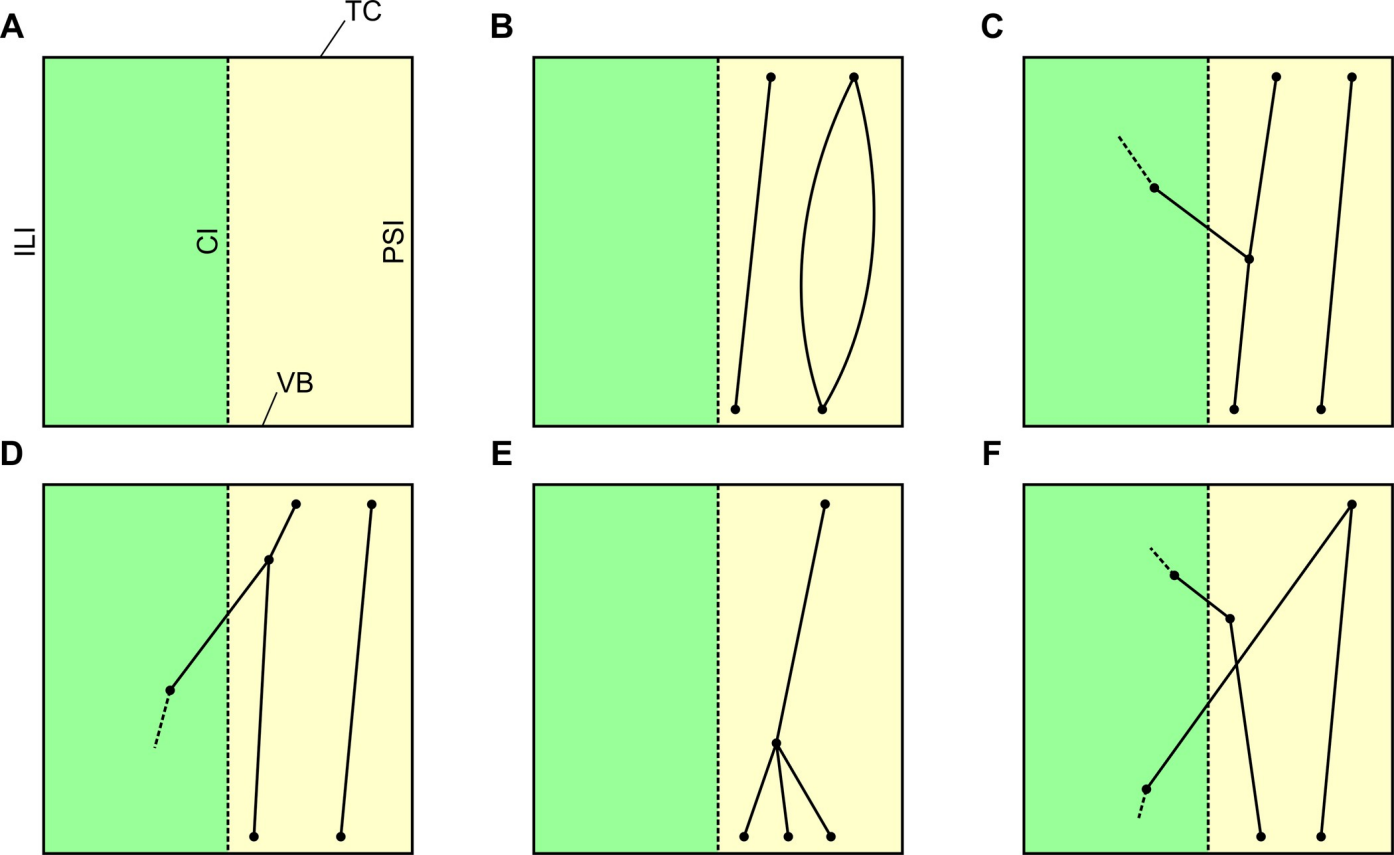
Consolidated topology of the paraseptal half of the NCTI. A description is given in Table 6. (A) Plain, (B) Simple, (C) Simple with crossover IL-PS, (D) Simple with crossover PS-IL, (E) Complex but no crossover, (F) Complex with crossover.

We found it suitable to consolidate topology of the paraseptal half of the NCTI into 6 different groups depending on its complexity (Table 6. and Fig 11). The inferolateral half of the NCTI is too complex and diverse to be consolidated into a reasonable number of topologies.

**Table 6:** Classification of network complexity in the paraseptal half of the NCTI.

Type	Description	#
Plain	no PM segments	2
Simple	one or more unbranched PM segments or parallel segments	13
Simple, with crossover IL-PS	same as Simple, but with one segment entering from inferolateral half of the NCTI	16
Simple, with crossover PS-IL	same as Simple, but with one segment leaving into inferolateral half of the NCTI	7
Complex, no crossover	branching and merging	2
Complex, with crossover	same as Complex, but one or more crossovers, either from paraseptal to inferolateral or vice versa	12

A graphical representation is shown in Fig 11. # … number of specimens the class was found in.

### Histology

**Fig 12:**
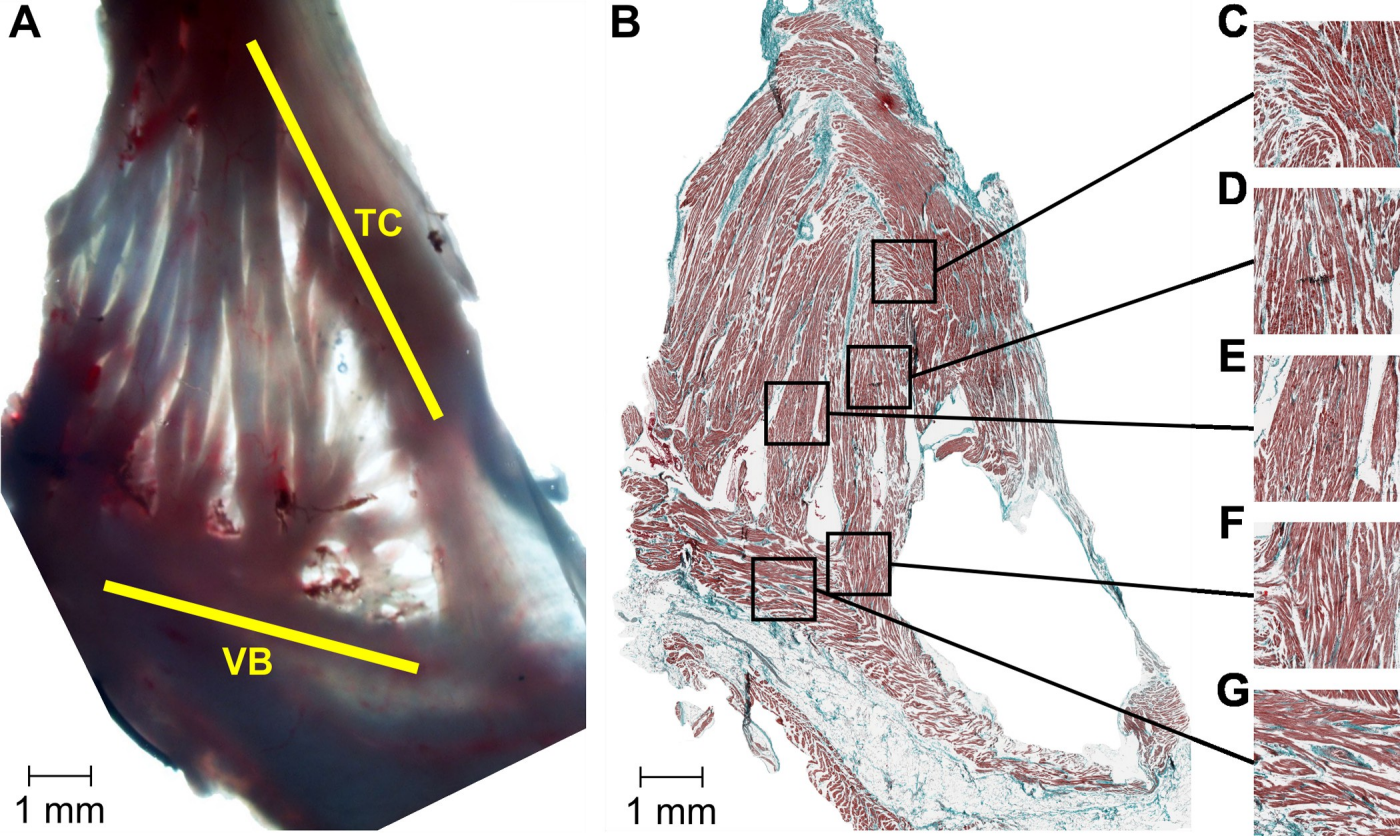
Histology of the CTI. (A) Macrograph showing a typical CTI structure. (B) Micrograph cut parallel to the CTI plane. (C) Ramification in the TC, i.e. an exit site from the TC into the PM network. (D), (E) Central section of PMs. (F) Merging site of two PMs and transition from the PM network into the VB. (G) Center of VB. (B) and (C) show densely packed and parallel oriented fibers, whereas (A), (D), and (E) reveal a highly complex microstructure. Extracellular signal recordings in these areas can be expected to be fractionated and of small amplitudes. TC … terminal crest, VB … vestibule.

Macrographs taken from a right atrium prepared for histological examination reveal a typical network of pectinate muscle strands between the TC and VB (Fig 12A). Micrographs cut parallel to the CTI plane give an insight into the additional complexity at a microscopic view of fibers and conduction pathways. Depending on the location within the CTI marked differences in arrangement of fibers, clefts between fibers, and embedded connective tissue can be found. The embedded connective tissue represents patchy, diffuse or compact local fibrosis, an indicator for potential structural arrhythmogenesis in human hearts [30].

In the central part of the CTI (Fig 12D and 12E) we find predominantly an arrangement of densely packed and parallel oriented fibers. At the proximal and the distal part (Fig 12C, 12F and 12G) particularly close to the TC and the VB we find fibers with a larger fraction of connective tissue, abrupt changes of fiber direction, and interlaced and crossing arrangements of the fibers. In consequence we can expect mostly large and unfractionated electrograms in the central part of the CTI whereas close to the TC and VB we will frequently find highly fractionated and low amplitude electrograms with multiple deflections.

## Discussion

This work introduces a novel scheme of nomenclature to describe the cavotricuspid isthmus and a bilinear transformation technique to compare structure and topology of CTI of different size and contour.

Morphology and topology in this work was derived from rabbit atria used in electrophysiological experiments in our lab. Rabbit models are quite common in basic arrhythmia research as they share a lot of characteristics similar to human hearts [31] and have a comparable effective size of the heart [32]. The macroscopical and structural arrangement of the CTI in rabbits shown here is comparable to human heart studies made in [10, 11, 26, 33]. In these studies, quantitative evaluation to the same extent as our analysis was not performed, but the figures show similar structures and our classification scheme should be applicable. An open question in this regard is if the paraseptal half of the NCTI in human hearts can also be consolidated to similar classes shown here for rabbit hearts. The length ratio of PSI to ILI in human atria was previously found to be 0.63 [8] and 0.80 [9] compared to 0.75 in our work. PSI was defined differently in [8], therefore the larger difference in ratio, but the overall CTI shape in human atria was similar to our findings, i.e. isthmus lengths decrease towards the septum. In [9] on the other hand PSI-to-ILI ratio is almost the same but CI was found to be shortest, a geometry we only found in 2 specimens. Nevertheless, CTI contours in our work resemble the overall human CTI geometry and scaling of results to human atria seems feasible.

The anatomy of the CTI is known to play a crucial role in sustaining atrial arrhythmias and therefore also in choice of interventional strategy [4, 34–36]. Although anatomy is well described [10, 37] there are still inconsistencies in nomenclature. In particular the paraseptal border of the CTI, which is also the base of the triangle of Koch, is not consistent in literature [8, 9, 38] and should be standardized. The classification into three sectors from posterior to anterior which divide the isthmuses into three equally long portions on the other hand is consistent but seems rather arbitrary. The nomenclature presented in this work aims to take functional arguments into consideration for the anatomical description of the CTI by defining sectors based on the location of PM ramifications as shown in Fig 3.

A limitation of the presented scheme for classification is that the anatomy is reduced to a 2-dimensional representation. This might be valid for thin tissues like the CTI, but is not adequate for tissues with pronounced 3-dimensional architecture like the trabeculations in the ventricles or even the right atrial appendage where PMs cross atop each other and ramifications might not be visible in 2D images. Nevertheless, also in the area of the CTI epicardial muscles fibers with different orientations are present. These epicardial fibers can connect the lower right atrium with the coronary sinus musculature or a remote atrial region of the CTI and should be taken into account if atrial isthmus ablation fails [39, 40]. Excitation spread of this type is a special case and we therefore think that a reduction of the CTI to a 2D geometry is sufficient at least in rabbit atria.

Morphometric analyses showed that inter-individual variations are remarkable and that a statistical description is hardly achieved. Even a lightweight rabbit may have a large CTI with a very complex network and thick muscle bundles. Nevertheless, with our suggested normalization of the CTI by bilinear transformation and the classification of PM segments based on their starting and endpoints, statistical analyses can be performed for these clusters of PM segments. This might serve as a basis for parametrization of rule-based computer models. One shortcoming of the applied preparation technique is that the atrium is not in its natural 3D arrangement but has to be flattened. Stretch applied to the atrium while fixing it onto a carrier affects the measured lengths and diameters of PM segments. Muscle fibers are sensitive to stretch and might go into contracture or rupture if they are not adequately stretched. During preparation we tried our best to preserve the *in-vivo* stretch and since our preparations were viable for several hours, we deduce that stretch was not completely off.

Our topological analyses show that the number of PM segments roughly doubles in the central section between levels *p* and *a*. This implies that there are two times more uncoupled pathways for excitation spread. Our hypothesis is that sustained arrhythmias are more likely the more uncoupled and asynchronized conduction pathways are present per area.

The individual PM segments have to deliver the electrical impulse from the TC to the VB. At ramifications, where PM segments split up or merge, the electrical excitation wavefront necessarily has to become more complex: At merging sites wavefront collisions arise and at branching sites source-sink mismatches with the concomitant reduction in conduction velocity and risk of unidirectional conduction block are potentially arrhythmogenic [16, 41, 42]. Parallel pathways of electrically uncoupled fibers can become a source of unidirectional block and induce reentrant waves in the CTI [43, 44]. In a previous work we have shown that individual strands of PM can be activated retrograde even in sinus rhythm [20], a fact that would allow reentrant activation in the PM network in the presence of conduction slowing. Such parallel segments are predominantly found in the central sector of the inferolateral part of the CTI making this region probably most susceptible for sustaining arrhythmias. Therefore, we suggest describing the network of PMs in the CTI in detail based on the location of ramifications between the TC and VB.

Histological analyses showed that not only the macroscopic structure is complex but also the microstructure. We have used histograms as shown here to build 2D models of parts of the CTI to predict the impact of macro- and microstructure on the waveform of extracellular potentials and on the local excitation spread [45, 46]. The configuration of extracellular potentials reflects both dimensions which impedes identification of the “true” arrhythmogenic substrates. Our hypothesis is that a complete ablation line across the CTI is not always necessary and interventional protocols based at least on the macroscopic anatomy of the CTI would be preferable. A conceivable approach would be the generation of a large number of computational models with artificial CTI geometries based on the statistics, ramification distribution and topology patterns shown here and using these models to select those geometries which reflect a given patient-specific activation pattern best, similar to the approach described in [47]. This would probably provide a hunch of the actual CTI geometry *in-vivo* and thus help adapting the interventional procedure.

Such artificial CTI geometries could also add realistic macrostructure of this region to state-of-the art computer models. 3D-geometry of atria in computer simulations is given in universal atrial coordinates (UAC, [48]) and the normalized coordinates presented in this work can easily be transformed into these UAC.

With this work we introduce a novel and consistent nomenclature of the CTI and provide a scheme for comprehensive description of the morphology and topology of the CTI which can be applied to human atria as well. The detailed description of the CTI presented in this work provides a basis for future scalable rule-based models for validation of *in-vitro* experiments and integration into state-of-the art computer models. This hopefully fathoms the understanding of isthmus-dependent flutter and fibrillation and results in improved personalized strategies for interventional procedures.

## Supporting information

S1 Fig Comparison of morphological parameters between New Zealand and domestic rabbits. The only statistically significant differences were found in weight (A), area of the VT (I) and diameter of the VT (J).
(TIF)
