# Supplementary figures and images for: Diversity and complexity of the cavotricuspid isthmus in rabbits: A novel scheme for classification and geometrical transformation of anatomical structures
Source: PLoS One. 2022 Mar 1;17(3):e0264625. doi: 10.1371/journal.pone.0264625 (PMC8887761; doi:10.1371/journal.pone.0264625)

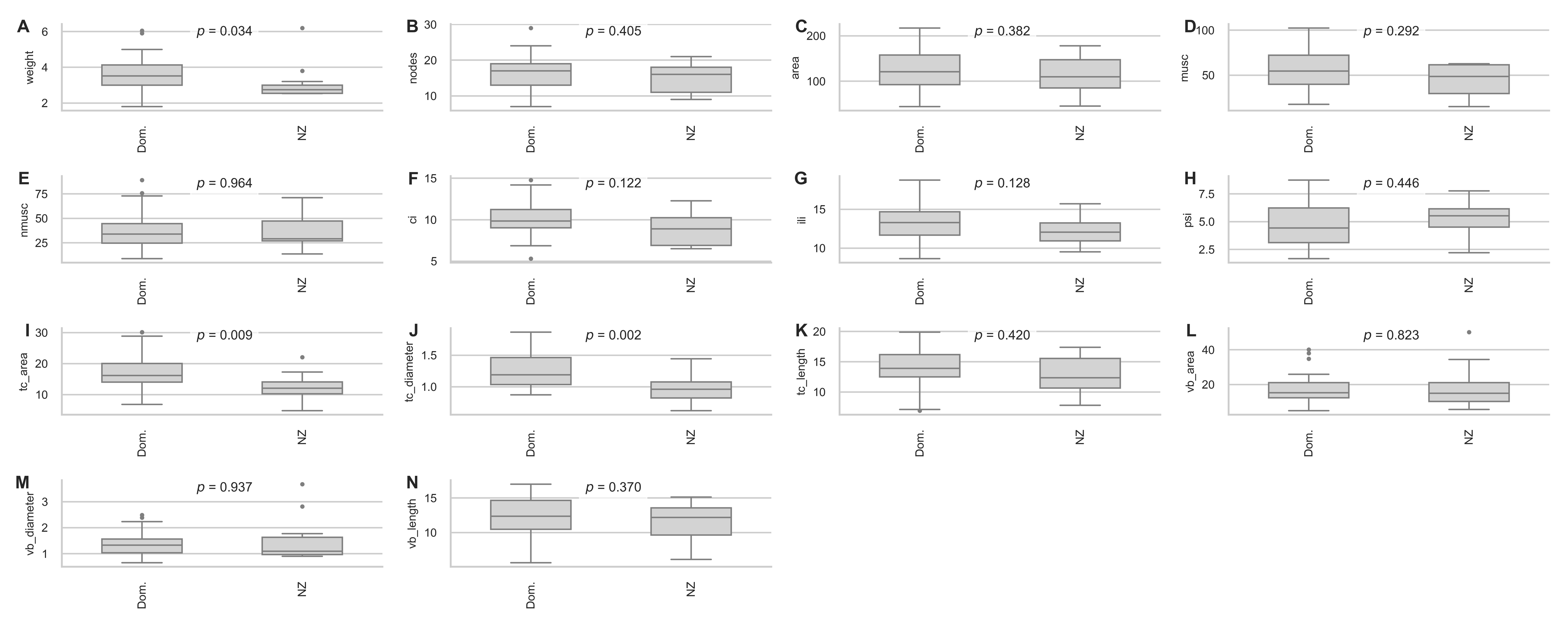

Supplement: S1 Fig — The only statistically significant differences were found in weight (A), area of the VT (I) and diameter of the VT (J). (TIF) [file pone.0264625.s001.tif]
